# On Cerebral, Spinal, and Ganglionic Paralysis

**Published:** 1856-04

**Authors:** 


*On Cerebral, Spinal, and Ganglionic Paralysis.* By Dr. Marshall Hall, F. R. S. (Lancet, Sept. 29, 1855.)

One great result has flowed from the investigation into the varied condition of the irritability of the muscular fibre in paralytic limbs—the fact that *hemiplegia* is sometimes *cerebral*, sometimes *spinal paralysis*—sometimes *limited* to the exclusion of the influence of the cerebrum, sometimes *extended* to the exclusion of the influence of the spinal marrow.

The distinction which I have established in regard to these two forms of paralysis, to which in this paper I add a third, is anatomical and positive.

When physicians speak of *hemiplegia,* they in reality use a term the signification of which has reference merely to a *symptom;* and that symptom may have a double or even a triple origin.

If hemiplegia affects and excludes the influence of the cerebrum only, the case is, I repeat it, cerebral paralysis; but if it affects or excludes the influence of the spinal marrow also, as it does in some severe cases, it is spinal paralysis; it will constitute one of those cases which, from our ignorance of their real nature, and from our error in viewing the terms cerebral paralysis and hemiplegia as synonymous and identical, have been regarded as *exceptional* cases.

These exceptional cases are rare amongst the milder cases of private practice; amongst the severer cases consigned to the workhouse, they may amount as in the subjects of Dr. Reynolds' inquiries, to three-fourths of the whole number of cases.

If our terms be once well defined, all ambiguity is removed; cerebral paralysis excludes the influence of the cerebrum only; spinal paralysis that of the spinal marrow also. The characteristics of each of these, when they are themselves distinct, are as fixed as the laws of physics.

To cerebral and spinal paralysis I must add a *third,* viz: *ganglionic* paralysis. This paralysis is excluded in pure cerebral paralysis; it is included in spinal paralysis.

Thus, in cerebral paralysis the muscles become *a*trophied; in spinal, in reality also ganglionic, paralysis, they become *heter*trophied, if for distinction, I may use that term. I have long regarded the ganglion on the posterior roots of the spinal nerves as parts of the true ganglionic system.

Thus, again, in cerebral paralysis the irritability of the muscular fibre is *augmented;* in spinal paralysis it becomes gradually more and more *diminished;* in ganglionic paralysis, if complete, it may become *extinct.*

In both an anatomical and in a physiological sense, the muscles in cerebral paralysis remain muscles, and their irritability, being unexhausted by the stimulus of volition, is, *pro tanto*, augmented, compared with that of the healthy limbs; whilst in spinal paralysis they gradually lose their muscular power, and in ganglionic paralysis they cease to be muscular, either in structure or in function. In certain cases, as M. Cruveilhier and M. Duchènne have shown, the muscular fibre undergoes the fatty degeneration which has recently attracted so much attention.

After these explanations and definitions, I think our investigations may proceed without any of those apparent exceptions and contradictions which have so much obstructed our progress. We must bear them continually in mind; and we must distinguish between true irritability and mere *force*, and the results will be uniform (unless, indeed, some other element of complication exist, still undetected); and all difference of opinion, so discreditable to physiological and medical science, will cease.

I will now, for the sake of still greater distinctness, throw the subject into a tabular form:

I.—*In Cerebral Paralysis*—

1. *The Reflex Actions,*
2. *The Influence of Emotion,*
3. *The Influence of Strychnine, and*
4. *The Irritability,*

*are more noticed in the paralytic than in the healthy limbs.*

II.—*In Spinal Paralysis*—

1. *The Reflex Actions,*
2. *The Influence of Emotions,*
3. *The Influence of Strychnine, are extinct, and*
4. *The Irritability diminished.*

III.—*In Ganglionic Paralysis*—

1. *The Structure, and*
2. *The Functions, may be alike destroyed.*

Cerebral paralysis may exist alone. Spinal paralysis, of course, *implies* cerebral paralysis. Ganglionic paralysis may exist with or without spinal muscular paralysis. In division or disease of the trifacial nerve we have ganglionic paralysis. And in a case which I formerly published, in which the digital nerve being injured, the nail ceased to grow as formerly. But as spinal paralysis implies cerebral paralysis, it also implies ganglionic paralysis. I have at this moment an interesting patient, who, from inflammation of the sciatic nerve from cold, has lost the power of the limb; the muscles are absolutely unaffectible by galvanism, atrophied, heterotrophied, and, I suppose, changed into fat. By restoring the healthy condition of the *nerve*, will the morbid change of structure undergo restoration? This is a question never yet agitated. It will require much observation and—*experiment*, to determine it satisfactorily; and I propose shortly to add to the present brief sketch some ample details.

I shall first add the enumeration of some other forms still to paralytic affection.—*Ranking's Abstract.*

